# Social acceptability of psilocybin-assisted therapy for existential distress at the end of life: A population-based survey

**DOI:** 10.1177/02692163231222430

**Published:** 2024-01-22

**Authors:** Louis Plourde, Sue-Ling Chang, Houman Farzin, Pierre Gagnon, Johanne Hébert, Robert Foxman, Pierre Deschamps, François Provost, Marianne Masse-Grenier, Jean-François Stephan, Katherine Cheung, Yann Joly, Jean-Sébastien Fallu, Michel Dorval, for the P3A Study Group

**Affiliations:** 1 Faculty of Pharmacy, Université Laval, Québec City, QC, Canada; 2 CHU de Québec-Université Laval Research Center, Oncology Division, Québec City, QC, Canada; 3 Lady Davis Institute for Medical Research, Jewish General Hospital, Montréal, QC, Canada; 4 Faculty of Medicine and Health Sciences, McGill University, Montréal, QC, Canada; 5 Faculty of Medicine, Université Laval, Québec City, QC, Canada; 6 Department of Health Sciences, UQAR, Lévis, Rimouski, QC, Canada; 7 CISSS of Chaudière-Appalaches Research Center, Lévis, QC, Canada; 8 Patient Partner, Montréal, QC, Canada; 9 Commission sur les soins de fin de vie, Montréal, QC, Canada; 10 School of Psychology, Université Laval, QC, Canada; 11 Institut universitaire en santé mentale de Montréal, Montréal, QC, Canada; 12 Department of Bioethics, New York University, New York, NY, USA; 13 Centre of Genomics and Policy, McGill University, Montréal, QC, Canada; 14 School of Psychoeducation, Université de Montréal, Montréal, QC, Canada; 15 Center for Public Health Research (CReSP), Montréal, QC, Canada; 16 Institut universitaire sur les dépendances (IUD), Montréal, QC, Canada

**Keywords:** Psilocybin, psychedelic, palliative care, end-of-life, psychological distress, survey, public opinion

## Abstract

**Background:**

Internationally, there is a growing interest in the potential benefits of psilocybin-assisted therapy to treat existential distress at the end of life. However, the social acceptability of this therapy is not yet well known.

**Aim:**

This study assesses the social acceptability of the medical use of psilocybin to treat existential distress at the end of life.

**Design:**

An online survey was conducted in Canada between November 23 and December 4, 2022. The questionnaire included items pertaining to perceptions, attitudes and concerns towards psilocybin-assisted therapy to treat existential distress at the end of life.

**Participants:**

The sample (*n* = 2800) was stratified by province, age and sex. Participants were adults from four provinces of Canada: Québec, Ontario, Alberta and British Columbia.

**Results:**

Overall, 79.3% considered psilocybin-assisted therapy a reasonable medical choice for a patient suffering from existential distress at the end of life, 84.8% agreed that the public health system should cover the costs of the intervention and 63.3% would welcome the legalisation of psilocybin for medical purposes. Previous psilocybin use (*p* < 0.0001, for all dependent variables), exposure to palliative care (*p* < 0.05, for all dependent variables) and a progressive political orientation (*p* < 0.05, for all dependent variables) were associated with more favourable attitudes towards psilocybin-assisted therapy at the end of life.

**Conclusion:**

The social acceptability of psilocybin-assisted therapy for existential distress at the end of life is rather high in Canada. These findings may contribute to efforts to mobilise resources and improve access to this emerging therapy in palliative and end of life care settings.

**What is already known on this topic?**

- There is a growing interest in psilocybin-assisted therapy worldwide, particularly to treat existential distress at the end of life.

**What this study adds?**

- In this study, we show that the social acceptability of psilocybin-assisted therapy to treat existential distress at the end of life is high in Canada and identify factors associated with favourable attitudes of the population towards it.

**How this study might affect research, practice or policy**

- Our findings may help mobilise resources to address barriers and challenges for implementing psilocybin-assisted therapy within palliative medicine and society. This could also have implications for policies regarding medical assistance in dying.

## Introduction

Over the last two decades, a growing number of clinical studies have shown the potential benefits of psychedelic substances in treating various, and often complex, mental conditions.^1–4^ Psilocybin, notably, was associated with a rapid and lasting reduction of the symptoms of existential distress in patients nearing the end of life.^5–7^

While these substances remain illegal in most jurisdictions worldwide, some have begun accepting their medical use. In January 2022, the Canadian government amended its Special Access Program to enable healthcare professionals to request a substance like MDMA or psilocybin to treat a patient with a serious or life-threatening condition, positioning the country at the forefront of such developments. The access is granted on a case-by-case basis if conventional therapies have been ineffective, are unsuitable for the patient or are unavailable.^8^

The increasing demand for psilocybin-assisted therapy raises many questions regarding access, safety, intervention protocol and training of healthcare professionals.^9,10^ However, its social acceptability, which has significant bearing on public policy and decision-making, is not yet well known. The objective of this study is to assess the social acceptability of psilocybin-assisted therapy in order to treat existential distress at the end of life and identify factors underlying perceptions, attitudes and concerns of the general population.

## Methods

### Study design and participants

A population-based, cross-sectional survey was conducted online. Participants were adults from four provinces in Canada: Alberta, British Columbia, Ontario and Québec. These provinces were selected as they account for > 86% of the Canadian population. Moreover, they are further along in having an established infrastructure for accessing psilocybin. The sample size was set a priori to 2800 and stratified by province, age and sex. The study was approved by the Research Ethics Committee of the CHU de Québec – Université Laval (2023-6501).

### Measures

The questionnaire was developed, revised and translated by an interdisciplinary team of researchers, and included 39 multiple-choice items and 2 open-ended questions. Definitions (e.g. Special Access Program, palliative care, existential distress and psilocybin-assisted therapy) were provided at the beginning of each section to ensure understanding among participants. Because of the adaptive nature of the questionnaire, not all respondents answered all items.

### Dependent variables

The first item of the questionnaire measured the awareness of the Special Access Program established by Health Canada, the main governmental health agency: ‘Have you heard about the Special Access Program?’. Other questions examined different facets of social acceptability towards psilocybin-assisted therapy: ‘To what extent would you agree that healthcare professionals should be allowed to administer psilocybin without going through Health Canada?’; ‘Do you think psilocybin is a reasonable choice for a palliative care patient suffering from existential distress?’; ‘In your opinion, should the public health system cover the costs of psilocybin-assisted therapy?’; ‘To what extent do you support the legalisation of psilocybin for medical purposes?’. Respondents were also asked to express their level of endorsement for three hypothetical scenarios on involving the use of psilocybin to address their own existential distress at the end of life: (1) within an assisted psychotherapy with a certified healthcare professional; (2) within a guided experience with a facilitator who is not a certified therapist; and (3) in a non-regulated context.

### Independent variables

Factors potentially associated with perceptions and attitudes towards psilocybin-assisted therapy included socio-demographic variables (province, age, sex, education level, household income and ethnicity), previous use of psilocybin (either in a therapeutic context, in a recreational context, in microdoses or any other way), exposure to palliative care (either as a palliative care patient, as a caregiver or volunteer, through someone close or in some other way) and political orientation assessed on a five-point Likert scale from ‘Conservative’ (right-wing) to ‘Progressive’ (left-wing).

### Data collection

A pre-test (*n* = 30) was conducted on November 17, 2022, to assess the questionnaire’s length, clarity and completeness. No significant modification was necessary. The questionnaire was then administered online between November 23 and December 4, 2022, by Léger (https://leger360.com/), a Canadian-owned survey and analysis firm with a panel of individuals having consented to being contacted for research. This was a closed survey. A link to the questionnaire, including an information and consent sheet (see Supplemental Material), both available in French and English, was emailed to potential participants. Participation was voluntary and anonymous.

### Data analysis

We used descriptive statistics to present the characteristics of the sampled population and to report proportions of participants for each dependent variable. To identify factors associated with the respondents’ attitudes, adjusted prevalence ratios and 95% confidence intervals were estimated using a generalised linear model with a log link and a Poisson working model. Models were mutually adjusted for all socio-demographic factors. Robust variances were obtained with sandwich estimators to account for the larger variance of Poisson variables compared with binomial variables. Since the point estimates and *p*-values were not materially different between weighted and unweighted analyses, only results of unweighted analyses are reported here. All analyses were conducted using SAS, version 9.4 (SAS Institute). Two-sided *p*-value <0.05 was considered statistically significant.

## Results

A total of 16,252 panel members were sent the invitation to participate in the survey, of whom 11,481 did not follow the link provided, 696 could not open the questionnaire because their stratum quota had been reached and 757 were ineligible. Of the 3318 eligible who accessed the survey web page, 170 (5%) refused to answer the questionnaire, and 348 (11%) did not complete it, resulting in a total of 2800 (84%) completed questionnaires that were analysed. Characteristics of the population surveyed are shown in Table 1. Because of the stratified sampling frame, sex and age groups were proportionally represented in the final sample. Overall, 19% of the surveyed population had previously used psilocybin-containing mushrooms, ranging from 15% in Québec to 26% in British Columbia.

**Table 1.** Multivariate regression model showing relationships between dependent variables and respondents’ characteristics.

Characteristic*s*	*n* (%)	Have you heard about the Special Access Program?			To what extent would you agree that healthcare professionals should be allowed to administer psilocybin without going through Health Canada?			Do you think psilocybin is a reasonable medical choice for a palliative care patient suffering from existential distress?			Currently, psilocybin-assisted therapy can be expensive. In your opinion, should the public health system cover the costs?			To what extent do you support the legalisation of psilocybin for medical purposes?		
		Yes vs. (No; I don’t know)			Strongly agree; Agree vs. (Neither disagree nor agree; Disagree; Strongly Disagree)			Yes, since it’s a personal choice; Yes, but only if other treatments have proven ineffective; Yes, for another reason vs. (No; Not sure)			Yes, fully; Yes, partially vs. (No)			Very favourable; Favourable vs. (Neither unfavourable nor favourable; Unfavourable; Very unfavourable)		
		%	aPR (95% CI)	*p*	%	aPR (95% CI)	*p*	%	aPR (95% CI)	*p*	%	aPR (95% CI)	*p*	%	aPR (95% CI)	*p*
Province																
Québec	1000 (35.7)	14.2	1.00	–	46.8	1.00	–	80.2	1.00	–	85.8	1.00	–	60.7	1.00	–
Ontario	600 (21.4)	17.6	1.23 (0.96–1.58)	0.1004	40.7	0.87 (0.76–0.99)	0.0324	80.9	1.01 (0.96–1.07)	0.7927	85.2	0.99 (0.95–1.04)	0.7934	64.1	1.06 (0.97–1.15)	0.2080
Alberta	600 (21.4)	24.2	1.70 (1.36–2.13)	0.0000	42.3	0.90 (0.80–1.02)	0.1053	78.2	0.98 (0.92–1.03)	0.3992	85.8	1.00 (0.95–1.05)	0.9912	67.0	1.10 (1.02–1.20)	0.0155
British Columbia	600 (21.4)	24.3	1.71 (1.37–2.14)	0.0000	45.8	0.98 (0.87–1.10)	0.6960	77.5	0.97 (0.91–1.02)	0.2399	82,0	0.96 (0.91–1.00)	0.0653	63.2	1.04 (0.96–1.13)	0.3361
Age (years)																
18–34	716 (25.6)	17.9	1.00	–	40.6	1.00	–	77.7	1.00	–	85.2	1.00	–	59.3	1.00	–
35–54	1050 (37.5)	19.0	1.06 (0.86–1.30)	0.5982	43.8	1.08 (0.95–1.22)	0.2325	77.1	0.99 (0.94–1.05)	0.7737	83.2	0.98 (0.93–1.02)	0.2828	60.3	1.02 (0.93–1.10)	0.7050
⩾55	1034 (36.9)	19.0	1.06 (0.85–1.33)	0.6023	47.3	1.17 (1.03–1.32)	0.0181	82.8	1.07 (1.01–1.13)	0.0252	86.3	1.01 (0.97–1.06)	0.5880	69.3	1.17 (1.08–1.27)	0.0003
Sex																
Female	1398 (50.0)	18.2	1.00	–	41.2	1.00	–	81.2	1.00	–	86.2	1.00	–	63.7	1.00	–
Male	1398 (50.0)	19.2	1.05 (0.89–1.24)	0.5533	47,0	1.14 (1.04–1.25)	0.0061	77.7	0.96 (0.92–1.00)	0.0369	83.7	0.97 (0.94–1.01)	0.1042	62.9	0.99 (0.93–1.05)	0.7084
Education																
No university degree	1584 (56.9)	17.3	1.00	–	44,0	1.00	–	79.4	1.00	–	87.1	1.00	–	63.3	1.00	–
University degree(s)	1200 (43.1)	20.6	1.19 (1.01–1.40)	0.0386	44.5	1.01 (0.91–1.11)	0.8223	79.2	1.00 (0.96–1.04)	0.9306	82.3	0.94 (0.91–0.98)	0.0022	63.3	1.00 (0.94–1.06)	0.9982
Income																
Less than $50,000	751 (29.7)	17.1	1.00	–	44.8	1.00	–	77.5	1.00	–	85.4	1.00	–	61.6	1.00	–
$50,000–$99,999	895 (35.4)	18.3	1.07 (0.87–1.33)	0.5235	42.4	0.95 (0.85–1.06)	0.3363	79.1	1.02 (0.97–1.07)	0.4600	83.3	0.98 (0.93–1.02)	0.2624	62.2	1.01 (0.94–1.09)	0.7979
$100,000 or more	881 (34.9)	20.5	1.20 (0.97–1.48)	0.0962	45.7	1.02 (0.91–1.14)	0.7233	81,0	1.04 (0.99–1.10)	0.1138	86,0	1.01 (0.96–1.05)	0.7542	65.7	1.07 (0.99–1.15)	0.1039
Ethnicity																
White	2257 (82.0)	19.5	1.00	–	46.2	1.00	–	80,0	1.00	–	85.4	1.00	–	65.4	1.00	–
Indigenous	60 (2.2)	17.3	0.89 (0.54–1.47)	0.6369	52.4	1.13 (0.89–1.45)	0.3140	76.6	0.96 (0.83–1.10)	0.5402	89.6	1.05 (0.96–1.14)	0.2734	67.4	1.03 (0.87–1.22)	0.727
Other	437 (15.9)	15.4	0.79 (0.61–1.02)	0.0692	34.4	0.74 (0.63–0.88)	0.0004	76,0	0.95 (0.89–1.02)	0.1337	81.2	0.95 (0.90–1.01)	0.0880	52.8	0.81 (0.72–0.90)	0.0001
Political affiliation																
Conservative	493 (20.3)	17.1	1.00	–	42.9	1.00	–	76.1	1.00	–	78.4	1.00	–	58.5	1.00	–
Neutral	866 (35.7)	15.7	0.92 (0.72–1.17)	0.4852	39.9	0.93 (0.82–1.06)	0.2836	74.2	0.98 (0.91–1.04)	0.4674	81.9	1.04 (0.99–1.11)	0.1458	55.5	0.95 (0.86–1.04)	0.2854
Progressive	1070 (44.0)	22.4	1.31 (1.06–1.62)	0.0136	48.6	1.13 (0.89–1.45)	0.0460	85.1	1.12 (1.06–1.19)	0.0002	90.3	1.15 (1.09–1.21)	0.0000	72.6	1.24 (1.14–1.35)	0.0000
Has used psilocybin																
No/no answer	2270 (81.1)	16.4	1.00	–	40.2	1.00	–	76.1	1.00	–	83.3	1.00	–	59.4	1.00	–
Yes	530 (18.9)	31.9	1.95 (1.65–2.29)	0.0000	64.9	1.62 (1.48–1.77)	0.0000	93.6	1.23 (1.19–1.28)	0.0000	91.4	1.10 (1.06–1.14)	0.0000	80.9	1.36 (1.28–1.44)	0.0000
Has been exposed to palliative care																
No	1535 (55.2)	15.9	1.00	–	42.4	1.00	–	75.9	1.00	–	82.5	1.00	–	61.2	1.00	–
Yes	1265 (44.8)	22.6	1.42 (1.21–1.67)	0.0000	46.5	1.10 (1.01–1.20)	0.0375	83.5	1.10 (1.06–1.15)	0.0000	87.7	1.06 (1.03–1.10)	0.0003	65.8	1.08 (1.01–1.14)	0.0170
		Global: 18.7%			Global: 44.2%			Global: 79.3%			Global: 84.8%			Global: 63.3%		

aPR: adjusted prevalence ratio; 95% CI: 95% confidence interval.

Globally, only 18.7% of respondents were aware of the Special Access Program enabling healthcare professionals to request psilocybin to treat a patient. Awareness varied significantly across provinces. University graduates, progressives, ever users of psilocybin, as well as those exposed to palliative care, were also more likely to have heard of the programme.

In terms of acceptability, 79.3% of Canadians considered psilocybin-assisted therapy a reasonable medical choice for a patient suffering from existential distress at the end of life, 44.2% agreed that healthcare professionals should be allowed to administer psilocybin without going through Health Canada, 84.8% agreed that the public health system should cover the costs of the intervention, either totally or partially and 63.3% would welcome the legalisation of psilocybin for medical purposes. Previous psilocybin use (*p* < 0.0001), exposure to palliative care (*p* < 0.05) and a progressive political orientation (*p* < 0.05) were positively associated with all indicators of acceptability, as observed for all dependent variables reported (see Table 1). Still, a majority of those who consider themselves politically conservative would support the legalisation of psilocybin for medical purposes. Respondents from ethnic minority groups tended to have less favourable attitudes compared to those who identified as white.

**Figure 1.**
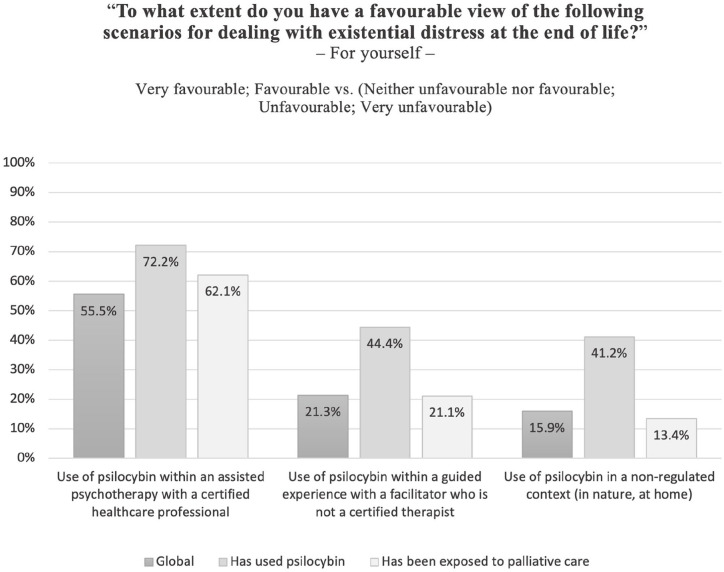
Percentage of respondents having a favourable view of psilocybin to treat existential distress at the end of life depending on the context of use.

As Figure 1 illustrates, 55.5% of respondents had a favourable view of psilocybin to treat existential distress at the end of life if used within the regulated context of psychotherapy, in sharp contrast with less regulated (21.3%) and non-regulated (15.9%) contexts.

## Discussion

### Main findings

In this peer-reviewed academic study on the social acceptability of psilocybin-assisted therapy at the end of life, we found that most Canadians have favourable attitudes about this emerging intervention. Those results are comparable to what has been observed in smaller population-based surveys conducted in the country and elsewhere.^11–14^ More than three out of four respondents considered psilocybin-assisted therapy a reasonable medical option to treat existential distress at the end of life, with the vast majority agreeing that the costs should be covered, at least partially, by the universal healthcare system.

Survey results did not vary significantly across provinces. Previous experience with psilocybin, exposure to palliative care and political orientation were the main factors associated with the respondents’ attitudes, followed by ethnicity and age.

### What this study adds?

Our findings are largely consistent with those of population-based surveys previously conducted in Canada,^11,12^ England^13^ and Australia.^14^ These surveys, mostly commissioned by interest groups or organisations, have not been published in scientific journals. Additionally, our study is novel as it focusses on treating existential distress at the end of life, an intricate condition for which treatment options are still limited and often ineffective.^15^

In light of the meteoric rise in the interest in psychedelic-based therapies, Canada’s experience with granting therapeutic psilocybin access could have far-reaching impacts, both locally and globally. Consequently, more research is required to address the many facets of implementing psilocybin-assisted therapy within medico-legal and ethical frameworks. As acceptability is high in this study, it may help stakeholders mobilise resources to address barriers and challenges.^16^ Since decision-makers and politicians are influenced by public opinion, results may also have a spillover effect on other contexts.^16^ The results obtained from this survey are timely in the wake of the growing worldwide interest towards psilocybin-assisted therapy. The implications of using this type of intervention to alleviate the existential suffering of patients nearing the end of life are manifest, as evidenced by a recommendation made in February 2023 by the Canadian Special Joint Committee on Medical Assistance in Dying, calling for an improvement of access to this therapy, as part of palliative care supports.^17^

### Strengths and limitations of the study

Our sample was large, and we obtained a high response rate. Measures were taken to follow the reporting guidelines set forth by the CHERRIES checklist.^18^ However, the present study shares the general limitations of web-based surveys, notably potential volunteer bias.^19^

Despite these limitations, our findings may contribute to efforts to mobilise resources and improve access to this emerging therapy in palliative and end of life care settings.

## Supplemental Material

sj-pdf-1-pmj-10.1177_02692163231222430 – Supplemental material for Social acceptability of psilocybin-assisted therapy for existential distress at the end of life: A population-based survey Click here for additional data file. Supplemental material, sj-pdf-1-pmj-10.1177_02692163231222430 for Social acceptability of psilocybin-assisted therapy for existential distress at the end of life: A population-based survey by Louis Plourde, Sue-Ling Chang, Houman Farzin, Pierre Gagnon, Johanne Hébert, Robert Foxman, Pierre Deschamps, François Provost, Marianne Masse-Grenier, Jean-François Stephan, Katherine Cheung, Yann Joly, Jean-Sébastien Fallu, Michel Dorval and for the P3A Study Group in Palliative Medicine

sj-pdf-2-pmj-10.1177_02692163231222430 – Supplemental material for Social acceptability of psilocybin-assisted therapy for existential distress at the end of life: A population-based survey Click here for additional data file. Supplemental material, sj-pdf-2-pmj-10.1177_02692163231222430 for Social acceptability of psilocybin-assisted therapy for existential distress at the end of life: A population-based survey by Louis Plourde, Sue-Ling Chang, Houman Farzin, Pierre Gagnon, Johanne Hébert, Robert Foxman, Pierre Deschamps, François Provost, Marianne Masse-Grenier, Jean-François Stephan, Katherine Cheung, Yann Joly, Jean-Sébastien Fallu, Michel Dorval and for the P3A Study Group in Palliative Medicine
