# Supplementary material for: Social acceptability of psilocybin-assisted therapy for existential distress at the end of life: A population-based survey
Source: Palliat Med. 2024 Jan 22;38(2):272–8. doi: 10.1177/02692163231222430 (PMC10865753; doi:10.1177/02692163231222430)
Supplement: sj-pdf-1-pmj-10.1177_02692163231222430 – Supplemental material for Social acceptability of psilocybin-assisted therapy for existential distress at the end of life: A population-based survey [file sj-pdf-1-pmj-10.1177_02692163231222430.pdf]

Participant Information Sheet

Project Title
Survey on the social acceptability of the use of psilocybin (“magic mushrooms”) for therapeutic purposes.

Principal Investigator
Michel Dorval, Ph.D.
Full Professor, Faculty of Pharmacy at Université Laval
Researcher at the CHU de Québec-Université Laval Research Center

Name of Funding Organisation
Fonds de recherche du Québec – AUDACE Intersectoral Program

Preamble
Psilocybin is the psychoactive element found in so-called “magic” mushrooms.

Clinical trials have shown that a single moderate dose of psilocybin combined with psychotherapy can significantly reduce anxiety and depressive symptoms over a long period of time in people with advanced cancer and suffering from existential distress at the end of life.

Although Canadian federal law prohibits the possession, production and sale of these mushrooms, a recent amendment to the Food and Drug Regulations now allows Canadian physicians to request special access to psilocybin for therapeutic purposes for their patients.

Study Objective
This research project aims to assess the social acceptability of using psilocybin (“magic mushrooms”) for therapeutic purposes.

Ultimately, the results of this research will contribute to formulating recommendations to government authorities to establish guidelines for the use of psilocybin for therapeutic purposes that will take into account the perceptions of the general population.

Study Conduct
The method used is a population-based survey by questionnaire. This questionnaire, which is conducted online, will collect the views of 2,800 people from the Canadian general population to obtain the most representative portrait possible of their beliefs, knowledge and attitudes toward the use of psilocybin ("magic mushrooms”) for therapeutic purposes.

Participation Modality
Your participation consists of answering an **online questionnaire of approximately 10 minutes**. You may receive one or two reminder emails after this mailing.

Your participation is entirely voluntary. **Completing the questionnaire constitutes consent to participate in the study.** You can choose not to answer certain questions, stop halfway or withdraw from the study at any time.

Your participation in this survey is important because it will allow obtaining a representative portrait and drawing valid conclusions on a subject where scientific data is scarce.

Confidentiality
When you submit your questionnaire, your answers will be automatically saved on a secure server of Léger Inc., the firm responsible for managing and transmitting the data to the research team. Only your answers will be saved. Your email address will not be passed on to the research team, which will have no way of identifying you.

All information collected will remain strictly confidential within limits set by the law. The data will only be used for research purposes in order to meet the scientific objectives of the project. This data will be kept for up to five years, after which it will be destroyed. Research data may be published in journals or be the subject of scientific presentations, but it will be impossible to identify you.

Contact Information
If you have any questions about the research project, you may contact the lead researcher, Professor **Michel Dorval, at (418) 682-8047** or the project coordinator, **Ms. Sue-Ling Chang, at (418) 682-7511, ext. 87838**.

If you have any questions about your rights as a participant in this research project, you can contact the Research Ethics Office of the CHU de Québec-Université Laval Research Center at (418) 525-4444 ext 52715. If you have any complaints or comments, you can contact the local Commissioner for Complaints and Quality of Services at the CHU de Québec at (418) 525-5312.
