# Supplementary material for: Social acceptability of psilocybin-assisted therapy for existential distress at the end of life: A population-based survey
Source: Palliat Med. 2024 Jan 22;38(2):272–8. doi: 10.1177/02692163231222430 (PMC10865753; doi:10.1177/02692163231222430)
Supplement: sj-pdf-2-pmj-10.1177_02692163231222430 – Supplemental material for Social acceptability of psilocybin-assisted therapy for existential distress at the end of life: A population-based survey [file sj-pdf-2-pmj-10.1177_02692163231222430.pdf]

Questionnaire on the social acceptability of the medical use of psilocybin in Canada (Plourde et al., 2023)

Background: In January 2022, Health Canada implemented a Special Access Program (SAP) allowing healthcare professionals to request access to a restricted drug like MDMA or psilocybin – which is contained in "magic mushrooms" – to treat a patient with a serious illness. Access to these substances, which are otherwise prohibited in Canada, is only considered when conventional therapies have failed, are unsuitable or unavailable in Canada. These requests are assessed on a case-by-case basis.

[Q1] Have you heard about the Special Access program?

[r1] Yes
[r2] No
[r98] I don't know

[Q2] If so, how did you hear about it? (Please check all that apply)

[r1] In the media (newspapers, magazines, television, radio)
[r2] On the Internet (websites, social networks, discussion forums)
[r3] From friends, family members or acquaintances
[r4] From a healthcare professional
[r5] In scientific publications
[r96] Other (please specify)

[Q3] Based on your current knowledge, what are the scientifically recognized benefits of psilocybin? (Please check all that apply)

[r1] Treatment of depression
[r2] Treatment of addiction
[r3] Treatment of existential distress caused by terminal illness
[r4] Treatment of anxiety
[r5] Treatment of chronic pain
[r6] Treatment of eating disorders
[r7] Treatment of degenerative diseases (Alzheimer)
[r8] Treatment of obsessive-compulsive disorder (OCD)
[r9] Treatment of post-traumatic stress disorder (PTSD)
[r10] Spiritual awakening / mystical experience
[r11] Increased creativity
[r12] Self-awareness
[r96] Other (please specify):
[r97] None of the above
[r99] I don't know / I prefer not to answer

[Q4] Based on your current knowledge, what are the potential risks associated with taking psilocybin?

Row:
[r1] Addiction
[r2] Physical injury (brain, organs)
[r3] Genetic damage
[r4] Heart problems
[r5] Overdose

[r6] Bad trip
[r7] Triggering of mental disorders
[r8] Risky behaviours
[r9] Cognitive impairments (memory, language, reasoning)
[r10] Panic

Column:
[c1] No risk
[c2] Low risk
[c3] Moderate risk
[c4] High risk
[c98] I don't know

[Q5] Have you ever taken psilocybin? (Please check all that apply)

[r1] Yes, in a therapeutic context
[r2] Yes, in a recreational context
[r3] Yes, in microdoses
[r96] Yes, another way (please specify)
[r5] No
[r99] I prefer not to answer

[Q5A] If so, how would you describe your experience(s) with psilocybin in general?

[c1] Very negative
[c2] Somewhat negative
[c3] Neither negative nor positive
[c4] Somewhat positive
[c5] Very positive

[Q6] Would you be open to using psilocybin for the first time or again?
(Please check all that apply)

[r1] Yes, to treat a physical problem
[r2] Yes, to treat a psychological problem
[r3] Yes, to explore my consciousness
[r4] Yes, to have a spiritual/mystical experience
[r5] Yes, to have fun
[r6] Yes, out of curiosity
[r96] Yes, for another reason (specify)
[r8] No
[r98] Not sure / I prefer not to answer

[Q6A] If not, why would you not be open to using psilocybin?
(Please check all that apply)

[r1] I'm afraid of being judged by my family.
[r2] I've had a bad experience with psilocybin (magic mushrooms).
[r3] I know someone who has had a bad experience with psilocybin (magic mushrooms).
[r4] The use of psilocybin is illegal.
[r5] The use of psilocybin could affect my physical health.
[r6] The use of psilocybin could affect my mental health.
[r7] I want to see more studies on psilocybin before I try it.
[r8] I'm afraid of having a bad trip.
[r9] I'm afraid of becoming addicted.
[r96] Other (please specify)
[r99] Not sure / I prefer not to answer

[Q7] Would you prefer psilocybin in synthetic form (tablet/capsule) or natural form (mushrooms)?

[r1] Synthetic
[r2] Natural
[r97] No preference

[Q8] Would you have a preference for a high, moderate, or microdose (below the threshold of the psychedelic experience) of psilocybin?

[r1] High
[r2] Moderate
[r3] Microdose
[r97] No preference

Background: The definition of palliative care adopted in this study is that of the World Health Organization. Palliative care is an approach that improves the quality of life of patients (adults and children) and their families who are facing problems associated with life-threatening illness. It prevents and relieves suffering through the early identification, correct assessment and treatment of pain and other problems, whether physical, psychosocial or spiritual.

[Q9] Have you had any exposure to palliative care? (Please check all that apply)

[r1] Yes, I'm in palliative care myself
[r2] Yes, as a caregiver or volunteer
[r3] Yes, through someone close to me
[r96] Yes, in some other way (please specify)
[r5] No

Definition: Existential distress at the end of life is a complex condition experienced by many individuals facing a terminal illness and/or a prognosis of impending death. This distress encompasses symptoms of depression, anxiety, demoralization and loss of meaning.

[Q10] To what extent do you think existential distress is a common condition in palliative care?

[r1] Very common
[r2] Somewhat common
[r3] Not very common
[r4] Not at all common
[r98] I don't know

[Q11] How much do you agree or disagree with the following statements?

Row:

[r1] Palliative care is a necessary response to the suffering of people who are seriously ill or at the end of life.

[r2] Palliative care allows people who are seriously ill to live as serenely as possible the end of their lives.

[r3] Palliative care allows people who are seriously ill or at the end of life to die with dignity.

[r4] Patients' existential distress is appropriately diagnosed and managed in the current palliative care setting.

Column:
[c1] Strongly disagree
[c2] Disagree
[c3] Neither disagree nor agree
[c4] Agree
[c5] Strongly agree

Background: Clinical studies have shown potential benefits of psilocybin in the management of existential distress in patients facing terminal illness. This type of therapeutic intervention typically involves only a single session assisted by a moderately high dose of psilocybin, preceded by one or more preparation session(s) and followed by one or more sessions with a psychotherapist who facilitates the integration of insights that have occurred during the experience.

[Q12] To what extent do you have a favourable view of the following scenarios for dealing with existential distress at the end of life?
Scenario 1: Use of psilocybin within an assisted psychotherapy with a certified healthcare professional
Row:
[r1] For yourself
[r2] For someone close to you
[r3] For others
Column:
[c1] Very unfavourable
[c2] Unfavourable
[c3] Neither unfavourable nor favourable
[c4] Favourable
[c5] Very favourable
Scenario 2: Use of psilocybin within a guided experience with a facilitator who is not a certified therapist
Row:
[r1] For yourself
[r2] For someone close to you
[r3] For others
Column:
[c1] Very unfavourable
[c2] Unfavourable
[c3] Neither unfavourable nor favourable
[c4] Favourable
[c5] Very favourable
Scenario 3: Use of psilocybin in a non-regulated context (in nature, at home)
Row:
[r1] For yourself
[r2] For someone close to you
[r3] For others
Column:
[c1] Very unfavourable
[c2] Unfavourable
[c3] Neither unfavourable nor favourable
[c4] Favourable
[c5] Very favourable

[Q13] To what extent would you agree that legal restrictions should be relaxed to facilitate access to psilocybin in a therapeutic setting?
[c1] Strongly disagree
[c2] Disagree
[c3] Neither disagree nor agree
[c4] Agree
[c5] Strongly agree

[Q14] To what extent would you agree that healthcare professionals should be allowed to administer psilocybin without going through Health Canada?

[c1] Strongly disagree
[c2] Disagree
[c3] Neither disagree nor agree
[c4] Agree
[c5] Strongly agree

[Q15] Do you think psilocybin is a reasonable medical choice for a palliative care patient suffering from existential distress? (Only one possible response choice)

[r1] Yes, since it's a personal choice
[r2] Yes, but only if other treatments have proven ineffective
[r96] Yes, for another reason (specify)
[r95] No (please specify)
[r97] Not sure

[Q16] If you wanted to use psilocybin therapeutically, which of the following means would seem the best way for you to access it?

[r1] From a healthcare professional requesting it from Health Canada
[r2] On your own, after obtaining an exemption from Health Canada
[r3] On you own, without obtaining an exemption
[r97] Not applicable

[Q17] In your opinion, if you were to choose psilocybin-assisted therapy, what would be the reaction of those around you, generally speaking?

[r1] Very unfavourable
[r2] Unfavourable
[r3] Neither unfavourable nor favourable
[r4] Favourable
[r5] Very favourable
[r6] Not sure

[Q18] If someone close to you in palliative care confided in you that he or she was considering psilocybin-assisted therapy, how would you react?

[r1] Very unfavourably
[r2] Unfavourably
[r3] Neither unfavourably nor favourably
[r4] Favourably
[r5] Very favourably
[r6] Not sure

[Q18A] What would change your opinion about the use of psilocybin for therapeutic purposes? (Please check all that apply)

[r1] The opinion of a healthcare professional
[r2] The opinion of someone close to me
[r3] Scientific consensus
[r4] Media coverage

[r5] Social acceptability
[r6] Testimonials from public figures
[r96] Other (please specify)
[r99] Nothing would change my mind

[Q19] Do you believe that scientific research into the therapeutic use of psilocybin, particularly in palliative care, should be encouraged?
[r1] Yes
[r2] No
[r97] Not sure

[Q20] Do you believe that healthcare professionals should experience psilocybin themselves as part of their training to assist patients in psychedelic therapies?
[r1] Yes
[r2] No
[r97] Not sure

[Q21] Currently, psilocybin-assisted therapy can be expensive. In your opinion, should the public system (RAMQ, Medicare) cover the costs?
[r1] Yes, fully
[r2] Yes, partially
[r3] No

[Q22] To what extent do you support the legalization of psilocybin for medical purposes?
[c1] Very unfavourable
[c2] Unfavourable
[c3] Neither unfavourable nor favourable
[c4] Favourable
[c5] Very favourable

[Q23] To what extent do you support the legalization of psilocybin for non-medical purposes?
[c1] Very unfavourable
[c2] Unfavourable
[c3] Neither unfavourable nor favourable
[c4] Favourable
[c5] Very favourable

[Q24a] In general, would you say your health is...?
[r1] Excellent
[r2] Very good
[r3] Good
[r4] Fair
[r5] Poor
[r98] I don't know
[r99] I prefer not to answer

[Q24b] In general, would you say your mental health is...?

[r1] Excellent
[r2] Very good
[r3] Good
[r4] Fair
[r5] Poor
[r98] I don't know
[r99] I prefer not to answer

[Q25] Using a scale of 0 to 10 where 0 means "Very dissatisfied" and 10 means "Very satisfied", how do you feel about your life as a whole right now?

[Q26] Which of these answers would best describe your general attitude toward drug use? (Only one possible response choice)

[r1] Drug use is mostly a private matter.
[r2] Drug use is mostly a public health issue.
[r3] Drug use is mostly a criminal issue.
[r96] Other (please specify)
[r99] No opinion

[Q27] Have you ever used or are you currently using the following substances?

Row:
[r1] Alcohol
[r2] Cannabis
[r3] Psychedelics (LSD, magic mushrooms)
[r4] MDMA (Ecstasy)
[r97] For quality control, please answer Regularly
[r5] Stimulants (Cocaine, speed)
[r96] Other drugs

Column:
[c1] Never
[c2] Once
[c3] A few times
[c4] Several times
[c5] Regularly
[c9] No response

[Q28] How important are your religious or spiritual beliefs to the way you live your life?

[r1] Very important
[r2] Somewhat important
[r3] Not very important
[r4] Not at all important
[r99] I prefer not to answer

[Q29] What is your political affiliation on the following scale?

[Q30] To which ethnic group(s) do you belong? (Please check all that apply)

[r1] Indigenous (First Nations, Métis or Inuit)
[r2] White
[r3] Black
[r4] Latin American
[r5] Arab
[r6] West Asian

[r7] South Asian
[r8] Southeast Asian
[r9] East Asian
[r96] Other (please specify)
[r98] I don't know
[r99] I prefer not to answer

[Q32] Which type of situation corresponds best to your current household composition?
[r1] Person living alone
[r2] Couple without children at home
[r3] Couple with child(ren) at home
[r4] Single-parent family
[r5] Couple or family with other persons (related or not)
[r6] Relatives only
[r7] Non-relatives only
[r96] Other situation
[r98] I don't know
[r99] I prefer not to answer

[Q33] What is the highest certificate, diploma or degree that you have completed?
[r1] Less than high school diploma or its equivalent
[r2] High school diploma or a high school equivalency
[r3] Trade certificate or diploma
[r4] College, CEGEP or other non-university certificate or diploma (other than trades certificates or diplomas)
[r5] University certificate or diploma below the bachelor's level
[r6] Bachelor's degree (e.g., B.A., B.Sc., LL.B.)
[r7] University certificate, diploma or degree above the bachelor's level
[r98] I don't know
[r99] I prefer not to answer

[Q34] What was your main occupation over the past 12 months?
[r1] Paid employment or self-employment
[r2] Attending school
[r3] Retired
[r4] Unemployed or looking for work
[r5] On maternity or paternity leave
[r6] On disability or sick leave
[r96] Other
[r99] I prefer not to answer

[Q35] What is your best estimate of the total annual income received by all household members, from all sources, before taxes and deductions?
[r1] Under $10,000
[r2] $10,000 to less than $20,000
[r3] $20,000 to less than $30,000
[r4] $30,000 to less than $40,000

[r5] $40,000 to less than $50,000
[r6] $50,000 to less than $60,000
[r7] $60,000 to less than $80,000
[r8] $80,000 to less than $100,000
[r9] $100,000 to less than $120,000
[r10] $120,000 to less than $140,000
[r11] $140,000 or more
[r98] I don't know
[r99] I prefer not to answer

[Q36] Do you accept to be contacted again for a future study on the same topic?
[r1] Yes
[r2] No

[Q37] What other issues do you think should be addressed in a research project or activity about psilocybin-assisted psychotherapies?

[Q38] What question(s) would you like to ask a healthcare professional about the therapeutic and/or psychological benefits of psilocybin?
